# Development of Novel Recombinant Antigens of Nucleoprotein and Matrix Proteins of *Porcine orthorubulavirus:* Antigenicity and Structural Prediction

**DOI:** 10.3390/v14091946

**Published:** 2022-09-01

**Authors:** Rocío Lara-Romero, José Luis Cerriteño-Sánchez, Susana Mendoza-Elvira, José Bryan García-Cambrón, María Azucena Castañeda-Montes, José Manuel Pérez-Aguilar, Julieta Sandra Cuevas-Romero

**Affiliations:** 1 Centro Nacional de Investigación Disciplinaria en Salud Animal e Inocuidad, Instituto Nacional de Investigaciones Forestales, Agrícolas y Pecuarias. Km 15.5 Carretera México-Toluca, Palo Alto, Cuajimalpa, Ciudad de México 05110, Mexico; 2 Posgrado en Ciencias de la Producción y de la Salud Animal, Facultad de Estudios Superiores Cuautitlán, Estado de México, Universidad Nacional Autónoma de México, Ciudad de México 04510, Mexico; 3 Maestría en Biología Experimental, Universidad Autónoma Metropolitana, Unidad Iztapalapa, Ciudad de México 09089, Mexico; 4 Facultad de Ciencias Químicas, Benemérita Universidad Autónoma de Puebla, Puebla 72000, Mexico

**Keywords:** *Porcine orthorubulavirus*, matrix protein, nucleoprotein, recombinant protein, *E. coli*

## Abstract

Blue eye disease (BED) is a swine viral infection that affects the pork industry of Mexico. *Porcine orthorubulavirus* (PRV) is the etiological agent, and the hemagglutinin-neuraminidase protein (HN) is characterized as the best antigen for serological tests, although other structural proteins, including the nucleoprotein (NP) and the matrix (M) protein, have been investigated during the infection of members of the *Paramyxoviridae* family, generating promising results. Herein, for the first time, we successfully produced and characterized both the NP and M proteins of PRV by using a recombinant strategy in the *E. coli* heterologous system. The ORF of the NP and M genes were cloned in-frame with the pET-SUMO expression vector. Recombinant proteins proved to be a sensitive target to detect seroconversion at 7 days until 28 days in vaccinated mice (BALB/c) by indirect ELISAs. Immunoreactivity was also tested using porcine serum samples, in which antibodies were recognized from early stages to a persistence of PRV infection, which is indicative that these proteins contain properties similar to native antigens. The predicted tertiary structure showed that both proteins have a conserved structure that resembles those found in others *Paramyxovirus*. Our results pave the way for developing biotechnological tools based on these proteins for the control and prevention of BED.

## 1. Introduction

*Porcine orthorubulavirus*, La Piedad Michoacán Mexico Virus (PRV-LPMV) [1], previously named *Porcine Rubulavirus*, is the etiological agent of blue eye disease (BED) in pigs. Similarly to other rubulaviruses, the genomic structure of PRV contains a single promoter in the untranslated 3’region, which is followed by the NP, P, M, F, HN, and L open reading frames (ORFs) that code for 10 proteins with structural, regulatory, and enzymatic functions [2]. The virion consists of a lipid bilayer or outer surface envelope derived from the plasma membrane of the host cell. Inserted into this bilayer are two glycoproteins: the fusion (F) and the hemagglutinin/neuraminidase (HN) [3,4]. Positioned right under the envelope is the matrix (M) protein, which is associated with part of the glycoproteins and the nucleocapsid. The nucleocapsid consists of a nonsegmented negative-sense single-stranded genomic RNA (approximately 15,000 nucleotides) that is tightly encapsulated by the nucleoprotein (NP). The P gene has the capacity to encode four possible polypeptides, namely, P, V, C, and I, from the same gene via editing and the alternative initiation of translation [5,6,7].

The first reports of BED were in La Piedad, Michoacán, Mexico, in 1980, and they were associated with respiratory, reproductive, and central nervous system disorders in swine. As a part of the lesions, the eyes showed a uni- or bilateral corneal opacity with a blue color in piglets [8,9]. Piglets are most susceptible to infection, showing high morbidity and mortality. The piglets often die within 2–7 days after the appearance of clinical signs. Clinical signs in adult pigs have mainly been associated with reproductive disorders, while mortality is lower than in piglets [9]. The pathogenesis of PRV depends on a positive organ tropism, associated with the receptors of sialic-acid-expressing cells and viral adhesion proteins [2]. After primary propagation in the respiratory tract and tonsils, the virus spreads throughout the brain via the trigeminal and olfactory nerves combined with low-level viraemia and passage through the immature blood–brain barrier. The virus has shown to be localized, and excretion occurs mainly via the respiratory tract and urine [10]. PRV infection has only been diagnosed in Mexico, and it is considered one of the most important diseases affecting the Mexican swine industry. It is an endemic disease in the central and western–central regions of Mexico (including the states of Michoacán, Guanajuato, Jalisco, and Estado de Mexico) with a seroprevalence ranging from 9 to 23.7% [11]. PRV diagnosis is difficult due to the variability in symptoms that are associated with cases with coinfections with other agents [12]. Clinical symptoms, necropsy findings, and histopathological changes can often provide insights into the etiology of the disease. Current methods used for the diagnosis of PRV infections in Mexico include serological tests and virus isolation [13,14,15]. The PRV has been shown to grow in many different cells, including pig kidney cells, with the characteristic of typical syncytial formations, demonstrating fusion activity [8]. Virus isolation, electron microscopy, direct immunofluorescence, classical RT-PCR, and real-time qPCR of P and NP proteins have been used for the detection of the infectious virus or the viral RNA of the PRV for various research purposes [15,16,17,18].

Since the first report of BED outbreaks, the pathogenesis of PRV has solely been based on the antigenicity and immunodominance analyses of the increase in HN proteins [19,20]. Nevertheless, recent studies suggested that the HN protein is not the only antigenic determinant participating in antigenic changes among different PRV strains [21].

For serological diagnosis, the most commonly used test is associated with hemagglutination inhibition due to the binding of the HN protein to the cellular receptor in the infected host. However, there is evidence that during PRV experimental infections in adult pigs, antibodies against the NP and the M proteins are also produced [22]. However, the antigenicity potential of these two proteins has not yet been fully investigated, even though the NP protein is highly conserved, and the most abundant viral protein is expressed in infected cells by PRV. Moreover, along the same lines, the M protein structure and sequence are also highly conserved among *Paramyxovirus*, with similar functions in the different members of the family; however, its antigenicity remains largely unknown [23,24,25]. In addition, studies of the genome organization and sequence homologies of the encoded proteins (i.e., NP, P, F, M, and L) as well as phylogenetic analyses revealed a high-sequence conservation in current circulating strains of the pig population compared to the original viral strain PRV-LPMV/1984 [26]. Hence, both proteins (i.e., NP and M) could serve as potential antigens in either vaccine development or serological diagnosis [27,28]. Thus, the overall objective of this study was to produce recombinant NP and M proteins in order to investigate in detail their antigenicity and immunogenicity to help in the diagnostic field. Lastly, the prediction of the quaternary structure of both NP and M proteins was also performed.

## 2. Materials and Methods

### 2.1. NP and M Proteins Prediction Structure and Amino Acid Alignment Comparison

Amino acid sequences from the NP and M proteins were analyzed to determine the relevant structural features and important molecular epitopes in the PRV reference strain (access number: BK005918). Different algorithms were utilized to predict the transmembrane regions of the protein, the potential antigenic sites and epitopes described by Kyte and Doolittle (1982) [29], Jameson-Wolf (1988) [30], and Kolaskar and Tongaonkar (1990) [31]. The probability that a given region lies on the surface of the protein was evaluated using the approach described by Emini et al. (1985) [32]. Molecular modeling was performed via the SWISS-MODEL server (Swiss Institute of Bioinformatics, Switzerland) using templates for the M protein and NP protein the X-ray structure of the Newcastle disease virus matrix protein (PDB accession code: 4G1G) [33] and the structure of the parainfluenza virus 5 nucleocapsid-RNA complex (PDB accession code: 4XJN), respectively [34]. The predictions of the protein structures were visualized and analyzed using the program PyMOL. In addition, to determine the conservation of the M and the NP proteins from PRV-LPMV, a sequence alignment comparison was performed, which includes other viral isolations and sequences from the *Paramyxovirus* family. Briefly, in the case of the NP protein, the sequence alignment was performed using 13 sequences (each composed of 519 amino acids) using the program MEGA X and the clustalW algorithm. An analysis of the percentage identity was performed using the NCBI blast tool. The sequences were taken from the NCBI Genbank database; similar protocols were used in the case of the M protein, but here, the alignment was performed using 14 sequences (each composed of 345 amino acids).

### 2.2. PRV NP and M Genes Amplification

The cDNA synthesis was carried out on RNA from PK15-infected cells by the reference strain, PRV-LPMV (access number: BK005918) [17], using the RevertAid First Strand cDNA Synthesis Kit, according to the manufacturer’s protocol (Invitrogen Life Technologies, Carlsbad, CA, USA). The amplification of full-length ORFs encoding the PRV nucleocapsid (NP) and matrix (M) protein was performed using PCR Master Mix 2x (Thermo Fisher Scientific, Waltham, MA, USA) and using the designed primers shown in Table 1. The PCR was performed as follows: 95 °C for 2 min; 35 cycles at 95 °C for 30 s; 72 °C for 60 s to extension. For the NP gene, primer alignment was conducted for 30 s at 65 and 60 °C for the M gene, followed by a final extension at 72 °C for 10 min. The resulting PCR products were purified from the gel using the Wizard^®^ SV Gel and PCR Clean-Up System Columns, according to the manufacturer’s protocol (Promega, Madison, WI, USA).

### 2.3. Bacterial Transformation with the Vector pJET 1.2/Blunt

The NP and M protein ORFs were used for the transformation of the *Escherichia coli* (*E. coli)* strain TOP10, which was applied as a cloning host, and the *E. coli* strain One Shot™ BL21 (DE3) cells, as the expression host (Invitrogen, Carlsbad, CA, USA) with MgCl_2_ and CaCl_2_. Briefly, for the preparation of competent cells, 25 mL of LB medium was inoculated with a BL21 (D3) colony and incubated at 37 °C while shaking (250 rpm) until the culture reached mid-log growth at a wavelength of 600 nm (0.35 of OD_600_), followed by incubation at 4 °C for 10 min. Then, cells were harvested by centrifugation (4000 rpm for 10 min) at 4 °C. The pellet was resuspended in 15 mL of a MgCl_2_-CaCl_2_ solution (i.e., 80 mM MgCl_2_ and 20 mM CaCl_2_) and centrifuged (4000 rpm for 10 min) at 4 °C. The cell pellet was suspended in 1 mL of 0.1 M CaCl_2_ solution, and 200 µL of competent cells was used for transformation by heat shock at 42 °C for 90 s using Plasmid pJET1.2/blunt (Thermo Fisher Scientific, Waltham, MA, USA) and 50 ng of purified DNA of the NP or M gene, respectively. Transformant cells were selected on LB agar plates supplemented with ampicillin (50 µg/mL) (LB-Amp). The bacterial plasmid was extracted using the Favor Prep Plasmid Extraction Mini Kit columns (Favorgen Biotech Corporation, Pingtung, Taiwan), according to the manufacturer’s instructions. The correct cloning of the ORFs of the NP- and M-positive colonies was verified using the PCR described previously. The resulting selected plasmids were designated as pJET-NP and pJET-M, respectively.

### 2.4. Cloning and Construction of the pET SUMO-NP and pET SUMO-M Plasmids

For cloning in the Champion™ pET SUMO expression vector (Thermo Fisher Scientific, Waltham, MA, USA), the selected positive bacterial plasmids (i.e., pJET-NP and pJET-M) were used as a template, and a modification was included in the final extension of the PCR for 30 min to place an adenine tail and to perform the ligation correctly. The purification of the PCR product was carried out at room temperature to avoid the degradation of the adenine tail and subcloning in the Champion™ pET SUMO vector. The colonies obtained were cultivated in 3 mL of LB-Amp medium at 37 °C for 16 h at 250 rpm for a subsequent extraction of plasmids in which it was evaluated to corroborate the correct orientation (5′-3′) of the ORFs; therefore, a PCR, as previously described, was carried out using a forward primer that hybridized in the high sequence of the expression vector (5′-AGATTCTTGTACGACGGTATTAG-3′) and as a reverse primer, which is the one described for the M and NP genes in Table 1. Recombinant plasmids were confirmed by sequencing with Sanger technology at the Biotechnology Institute of UNAM. The plasmid of a positive 5′-3′ colony was selected for the transformation of One Shot BL21 (DE3) cells, following the manufacturer’s recommendations (Invitrogen, Carlsbad, CA, USA), on an LB agar added with kanamycin (50 µg/mL) (LB-KA). Positive colonies were selected to carry out the expression test.

### 2.5. Expression and Purification of the Recombinant NP and M Proteins

For the expression of recombinant NP and M proteins, a colony from an LB-KA agar plate was inoculated into 3 mL of LB-KA and incubated in a bacterial shaker (250 rpm) at 37 °C for 12 h. Then, a second culture of 10 mL of LB-KA medium was inoculated with 100 microliters of previous bacterial culture and induced with 1.5 mM/L of isopropyl b-D-1-thiogalactopyranosid (IPTG) (Merck KGaA, Darmstadt, Germany) when the optical density of the culture reached between 0.5 and 0.6 OD_600 nm_, followed by incubation for an additional 6 h at 37 °C in a bacterial shaker. Induced cells were recovered from the medium by centrifugation (4000 rpm for 10 min), and supernatants and pellets were boiled for 10 min in a reducing sample buffer (0.0625 M Tris-HCl, 2% SDS, 10% v/v glycerol, 1.25% 2-mercaptoethanol, 0.01% bromophenol blue, and pH 6.8) and analyzed by 12% SDS-PAGE and visualized by Coomassie Brilliant Blue G-250 stain [35,36]. Subsequently, for purification, 100 mL of induced bacterial cultures was aliquoted into 50 mL centrifuge tubes and pelleted by centrifugation (4000 rpm for 10 min). The pellets were washed two times: first, with 50 mL of distilled water, centrifuged and pelleted at 4000 rpm for 10 min (4 °C), and then with 2% sucrose as indicated above. The pellets were resuspended with 400 mL of 0.1 M Tris-HCl buffer (5 mM, pH 7.5) and disrupted by mechanical rupture with a Gaulin Laboratory Homogenizer at 550 kg/cm for 15 min (APV Homogenizer Group, Wilmington, MA, USA). The inclusion bodies (insoluble phase) obtained by centrifugation (4000 rpm for 20 min, 4 °C) were washed with 50 mL of 1:1 water/1% Triton X-100 and recovered by centrifugation at 4000 rpm for 20 min (4 °C). The inclusion bodies were solubilized with 7% N-lauroylsarcosine sodium salt, 50 mM Tris-HCl buffer at pH 8, with agitation at 250 rpm for 12 h at 25 °C. The solubilized inclusion bodies of recombinant proteins were purified using 5 mL of HiTrap^®^ Chelating High Performance (GE Healthcare, Chicago, IL, USA) according the manufacturer’s instructions. The purified recombinant proteins were dialyzed versus the Tris-HCl 5 mM pH 8 buffer. The protein concentration was determined according to the Bradford methods (1976), resolved on SDS-PAGE gel, and transferred onto PVDF membranes (Merck Millipore, Darmstadt, Hesse, Germany) in order to be analyzed by WB. Briefly, the membranes were blocked with 5% nonfat milk in TBS-Tween buffer (20 mM Tris-HCl, pH 8, 0.15 M NaCl, and 0.05% Tween 20) at 4 °C for 16 h with moderate agitation. The blocked membranes were washed with TBS-Tween buffer and incubated with different antibodies; anti-histidine (diluted 1:5000) or a polyclonal swine serum sample (1:500) as the primary antibody, respectively, and a mouse anti-IgG conjugated to horseradish peroxidase (dilution 1:5000) or anti-pig IgG+ horseradish peroxidase (HRP)-conjugated as the secondary antibody (1:5000), respectively. Protein bands were visualized with DAB™ substrate (3,3′-diaminobenzidine tetrahydrochloride) (Sigma-Aldrich, St. Louis, MO, USA) with 10 mL of development solution (PBS, 12 mg of DAB, and 300 µL 3.4% H_2_O_2_).

### 2.6. Antigenicity of NP and M Recombinant Proteins by Mice Immunization

BALB/c mice were immunized at 21 old days with recombinant proteins NP and M. Five experimental groups were included: one negative control group immunized with sterile PBS (*n* = 8); one immunized group with recombinant NP protein (without adjuvant) (*n* = 8); one immunized group for recombinant M protein (without adjuvant) (*n* = 8); one control group immunized with the NP protein plus adjuvant (*n* = 8); one control group immunized with the M protein plus adjuvant (*n* = 8). Briefly, the immunization and bleeding scheme was as follows: All mice were inoculated into a fold of skin in the neck with a volume of 0.2 mL, and two doses at 0 and 14 days were administrated. The protein dose was 5 µg plus PBS or 5 µg of adjuvant AbISCO-100 (Isconova AB, Uppsala, Sweden) per mouse; blood samples were obtained by tapping the tail on days 0, 7, 14, 21 and 28. The animals were euthanized according to NOM-062-ZOO-1999. The protocol for the use of laboratory animals was previously approved by the INIFAP internal committee, previously approved under a permit from the IACUC (Institutional Animal Care and Use Committee), CENID-SAI, INIFAP. All procedures were in accordance with the Mexican legislation (NOM-062-ZOO-1999; SAGARPA), based on the Guide for the Care and Use of Laboratory Animals, NRC.

To evaluate the antigenicity of the recombinant proteins (i.e., NP and M), an indirect ELISA test was carried out using the serum samples obtained from the mice; briefly, 100 ng of purified protein was absorbed per well in a microplate and subsequently incubated with mice sera samples (diluted to 1:150). A secondary antibody (mouse anti-IgG-HRP) was used with a dilution of 1:5000. The chromogenic reaction was developed using 3,3′,5,5-tetramethylbenzidine (TMB) substrates, as previously described [35,36], and measured at an absorbance of 450 nm.

Statistical analyses of the results were performed using two-way ANOVA to compare immunized groups with protein plus adjuvant versus protein alone on different days. Differences at *p* < 0.05 were considered statistically significant with a 95% confidence interval, (* *p* < 0.05; ** *p* < 0.005; *** *p* < 0.0005) and graphs were constructed using SigmaPlot version 12.5 (Systat Software Inc., San Jose, CA, USA). All summary data are presented as means ± Standard Error of Mean (SEM).

### 2.7. Immunoreactivity of the NP and M Recombinant Proteins with Pig Serum Samples

For this assay, twelve pig sera samples from experimentally and naturally PRV-infected pigs were provided by a research project at the National Research Center, INIFAP (Instituto Nacional de Investigaciones Forestales, Agrícolas y Pecuarias) in Mexico City. Briefly, for experimentally infection; piglets (6 days of age) were infected by intranasal instillation with the PRV/PAC3/1992 (EF413173) low-virulence strain, provided by the Departamento de Microbiología e Inmunología, Facultad de Medicina Veterinaria y Zootecnia, UNAM, Mexico. The viral titer was determined by the calculation of 50% endpoint by serial dilution, and the titer of virus stock was 1 × 10^6^ TCID_50_/mL. Post-infection, the piglets were evaluated daily by a veterinarian, and clinical scores were observed and written down. The animals were handled at the house facility at the National Microbiology Research Centre (INIFAP), and they were fed with a commercial diet twice daily, having free access to water. All procedures followed the Mexican legislation (NOM-062-ZOO-1999; SAGARPA) based on the Guide for the Care and Use of Laboratory Animals, NRC. The experiment was previously approved under a permit from the IACUC (Institutional Animal Care and Use Committee), INIFAP. The serum samples collection consisted of six sera per day from different experimentally infected piglets (10 days old) at 5, 6, 10, 14, 30 and 75 days post-experimental infection; six serum samples from different natural PRV-infected pigs with persistent infection (>1 year) and blue eye as sequel, from a farm (Guanajuato, Mexico) with a history of sporadic outbreaks of BED. All sera were analyzed by Western blot tests using 250 ng of purified protein to determine the recognition capacity of the antibodies against the recombinant proteins NP and M. The PRV hyperimmune pig serum (75 days post-infection) previously tested by ELISA was used as a positive reference control. The WB results were categorized by comparing the pixel intensity between positive control and serum samples with ImageJ program [37] as follows: high grade had a four mark (+ + + +) recognition; +++ (moderate); ++ (slight); + (light); pig serum as a negative control with one negative mark (-).

## 3. Results

### 3.1. Tertiary Structure of the NP and M proteins and Amino Acid Alignment Comparison

**Figure 1:**
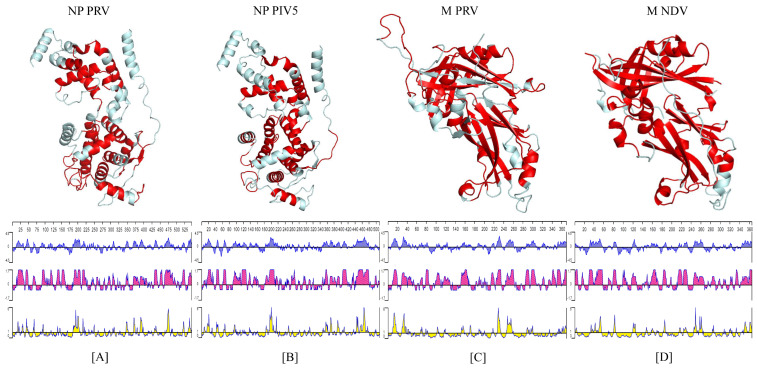
Tertiary structure of the NP and M proteins. Top panel: the structure of the PRV-NP monomer (**A**); PIV5-NP monomer (**B**); PRV-M monomer (**C**); NDV-M monomers (**D**) are displayed with epitopes colored in red. Bottom panel: Kyte and Doolittle hydrophobicity plot (blue: positive values indicate hydrophobic regions); Jameson–Wolf antigenic index (pink); Emini surface accessibility prediction (yellow).

The PRV-NP protein had a high sequence identity (58.82%) with the nucleoprotein parainfluenza virus 5 (PIV5-N); therefore, the same subdomain separation of the latter was utilized in the former. The different subdomains were the Narm segment consisting of the first 31 a.a, the NTD segment spanning from residues 32 to 262, the CTD segment from residues 263 to 371, and the Carm segment ranging from residues 372 to 401. The NTD and CTD subdomains have a significant role in forming the core of the promoter, while Narm and Carm segments seem to be involved in interfacial interactions with other protomers. We found 22 antigenic determinants in the PRV-NP protein that were mainly distributed along the NTD and CTD subdomains. As expected, based on their interfacial positions, the Narm and Carm subdomains did not seem to contain relevant information regarding possible epitopes. The identified epitopes were located in segments with high helical content. In the case of the PIV5-N protein, we found 19 antigenic determinants distributed similarly, as in the case of the PRV-NP (Figure 1A,B panel). Interestingly (and expected), the PRV-NP protein contained very low hydrophobic contents (blue plots in Figure 1) along the regions identified as antigenic regions, which were mainly distributed in the NTD and CTD domains (pink plots in Figure 1). Moreover, two regions identified as highly surface exposed were located in the NTD and the CTD subdomains (yellow plots in Figure 1). Finally, similar results were found in the case of the PIV5-N protein but with higher scores and more regions highly exposed to the protein surface (Figure 1A,B below). On the other hand, the PRV-M protein had a 25.27% sequence identity with the sequence of the Newcastle disease virus matrix protein (NDV-M); hence, the PRV-M structure could be similarly separated into two domains as in the case of NDV-M. Here, the N-terminal domain extended from residues 1 to approximately 186, while the C-terminal domain ranged from residues 191 to 369. These similar structures consisted of several β-sheets segments that were flanked by some α-helices. Most of the 14 antigenic determinants found in the PRV-M protein were positioned in segments with high β-sheet content in these two domains. A similar antigenic distribution was found in the case of the NDV-M protein with only 12 antigenic determinants (Figure 1C,D). Both PRV-M and NDV-M proteins displayed segments with very low hydrophobic content (blue plots in Figure 1) overlapping the antigenic regions. These regions were clearly distributed along the N- and C-terminal domains (pink plots in Figure 1). Lastly, both proteins had close to two regions with highly surface exposed protein regions in the N- and C-terminal domains (Figure 1C,D, yellow plots).

**Figure 2:**
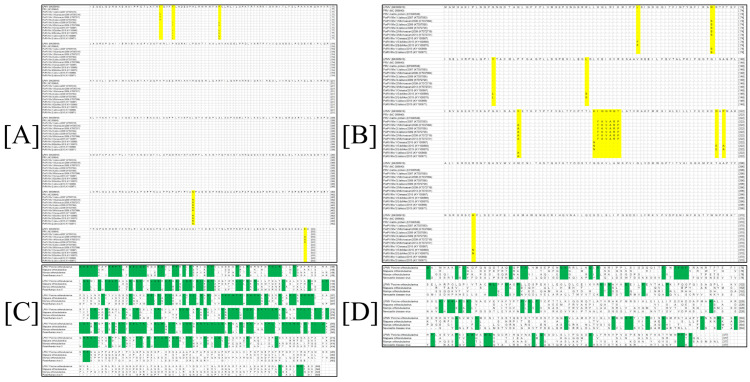
Multiple sequence alignment comparisons of the M and the NP proteins from PRV-LPMV. (**A**) Alignment for the PRV-LPMV NP protein with others viral isolations. (**B**) Alignment for the PRV-LPMV M protein with others viral isolations. (**C**) Comparison between the PRV NP protein and various members from the *Paramyxovirus* family. (**D**) Comparison between the PRV M protein and various members from *Paramyxovirus* family. Changes in amino acid identities into the protein systems are shown in yellow, while conserved regions are highlighted in green.

The amino acid alignment comparison showed that a sequence identity resulted in values ranging from 100% to 99.44% between the PRV NP protein and the different viral isolates reported in GenBank database; for the case of the PRV M protein, it produced values from 100% to 97.846% (Figure 2A,B). The alignment of both PRV proteins with different *Paramyxovirus* (Parainfluenza 5, Mumps and Mapuera virus) sequences resulted in several conserved regions located principally at the N-terminus of NP protein. The analysis resulted in the identification of 196 conserved sites through the length of the regions ranges from one to eight amino acid residues. As for the M protein, several conserved regions, distributed along the entire protein sequence, were identified and resulted in 56 conserved sites (Figure 2C,D).

### 3.2. Construction of an Expression System for NP and M Proteins

**Figure 3:**
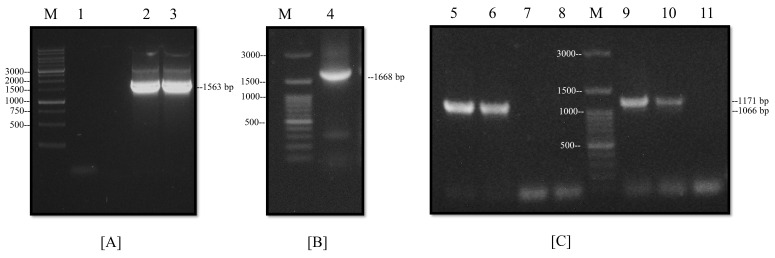
Construction of an expression system for NP and M proteins. (**A,B**) Analysis of the cloned NP gene by PCR: (1) negative control, (2–3) amplification of NP gene from pJET-NP, and (4) amplification of NP gene in-frame cloning with the pETSUMO vector. (**C**) Analysis of the cloned M gene by PCR: (5–6) amplification of the M gene from pJET-M, (7–8) negative controls, (9–10) amplification of the M gene in-frame cloning with the pETSUMO vector, (11) negative control, and (M) molecular weight marker.

The ORFs of the NP and M proteins were amplified from the pJET-NP and M vectors. The PCR products were analyzed on agarose gels to corroborate adequate amplifications at the expected molecular weights (i.e., 1563 and 1066 bp, respectively). Then, the PCR products were cloned into the pETSUMO expression vector. This plasmid allowed for the insertion of the gene in one-step cloning through A’, and that was generated by the Taq polymerase. After the ligation of the M and NP genes, the correct orientations of the inserts into the recombinant plasmids were analyzed by the PCR product with 1668 bp for the pETSUMO-NP vector and 1171 bp for the pETSUMO-M vector (Figure 3A,C). All tests and subsequent sequencing validated the expected results. The expression system produced recombinant proteins fused not only to 6-his tags to facilitate affinity purification but also to SUMO tags to increase protein solubility (all of which were under IPTG induction).

### 3.3. Expression of the PRV-NP and M Proteins in E. coli

**Figure 4:**
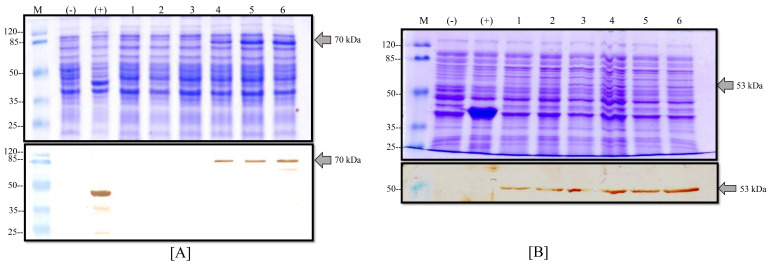
Expression of M and NP proteins in *E. coli*. (**A**) Production of the NP protein into the BL21-M strain: (above) Coomassie stain and (below) Western blot with anti-his antibodies of different clones induced with IPTG; (M) molecular weight marker, (-) negative control of BL21 strain, (+) positive control of BL21-CAT, and (1–6) different clones of BL21-NP. (**B**) Production of the M protein into the BL21-M strain: (above) Coomassie stain and (below) Western blot with anti-his antibodies of different clones induced with IPTG; (M) molecular weight marker, (-) negative control of BL21 strain, (+) positive control of BL21-CAT, and (1–6) different clones of BL21-M.

The transformed BL21 (D3)-competent cells with the pETSUMO vector were selected to produce NP and M recombinant proteins using IPTG as an inductor into LB medium. After 12 h of expression, induced cells were collected for analysis via SDS-PAGE Coomassie staining and Western blot. As shown in Figure 4, some clones analyzed for the NP protein did not produce proteins at the expected molecular weight possibly due to the fact of incorrect transformation. However, other clones produced an overexpression protein band at the expected molecular weight (71 kDa) that was subsequently confirmed by WB, resulting in a clear band (Figure 4A). On the other hand, other different clones of BL21-M protein were analyzed by SDS-PAGE showing a negligible increase in the protein band at the expected molecular weight (53 kDa) in the conventional Coomassie staining process compared to the positive control (BL21-CAT that produced a recombinant protein of 37 kDa). The WB analyses confirmed the presence of the recombinant proteins with different grades of production (Figure 4B). All controls displayed expected results. With this assay, the production of the NP and M proteins was confirmed in the *E. coli* expression system, and the strains were named BL21-PRV-NP and BL21-PRV-M, respectively.

### 3.4. Purification of the NP and M Recombinant Proteins

**Figure 5:**
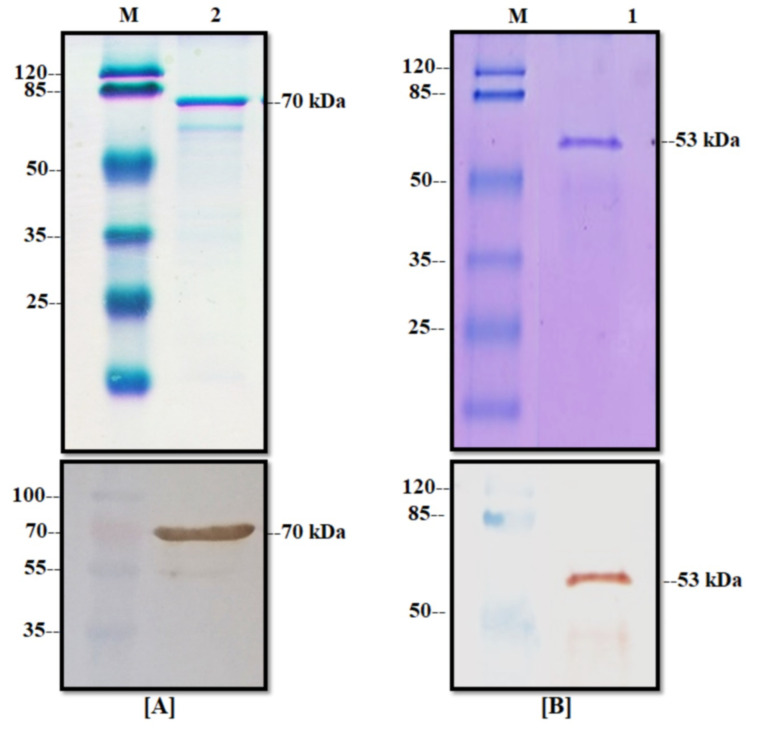
Purification of the NP and M proteins. (**A**) Elution of NP protein from affinity chromatography: (above) Coomassie stain and (below) Western blot with anti-his, (M) molecular weight marker, and (2) elution phase from column. (**B**) Elution of the M protein from affinity chromatography: (above) Coomassie stain and (below) Western blot with anti-his, (M) molecular weight marker, and (1) elution phase from column.

The produced recombinant proteins were purified from the insoluble fraction (inclusion bodies) because we observed that the major production was in this phase (data not shown). Inclusion bodies were recovered from cell pellets by mechanical rupture and dissolved into a sarcosyl buffer solution. The dissolved inclusion bodies were charged into a Ni-NTA agarose resin column and eluted with imidazole. The elution of the purification process was analyzed by SDS-PAGE Coomassie staining and the WB technique (Figure 5). As shown in Figure 5A, the NP protein presented a clear band with a high protein concentration in the elution phase with an average protein yield of 150 µg/mL from 100 mL of culture. In the case of the M protein, there was a band with the expected molecular weight of 53 kDa (Figure 5B) with a high degree of purity in the Coomassie stain. The average yield of this process for the M protein was 70 µg/mL of purified recombinant protein from 100 mL of culture. Finally, these one-step purified proteins were confirmed by WB analysis and by the presence of a signal for the recombinant proteins at the expected molecular weight.

### 3.5. Antigenicity Assays

**Figure 6:**
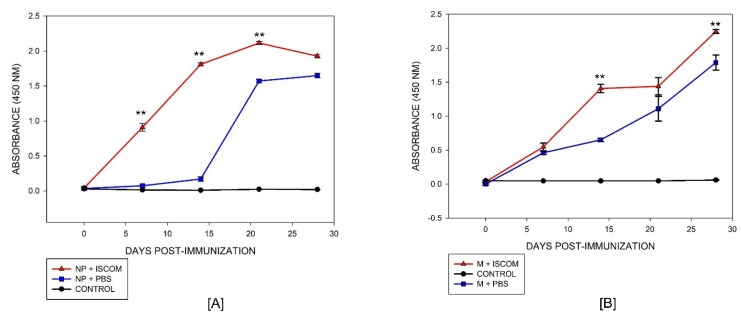
Antigenicity of NP and M proteins. (**A**) Indirect ELISA for the NP and (**B**) M proteins measured from antibodies produced in mice; (--▲--) antibodies from NP and M proteins produced with adjuvant, (--■--) antibodies from the NP and M proteins without adjuvant, and (--●--) negative control of non-immunized animals. Two-way ANOVA was used to compare immunized groups with protein plus adjuvant versus protein alone on different days. Differences at *p* < 0.05 were considered statistically significant with a 95% confidential interval (** *p* < 0.005).

Purified proteins were used to immunize mice, and antibody production was measured by indirect ELISA. As shown in Figure 6, both proteins produced specific antibodies with similar absorbance values at the end of the immunization. The antibody response of the NP protein displayed the capacity to induce an immune response at 7 days post-immunization, lasting until the end of the experiment (day 28), and it was significantly higher (*p* < 0.001) than the response of mice immunized with the NP recombinant protein alone, i.e., without an adjuvant (Figure 6A). In the case of the M recombinant protein, the antibody’s response increased after the second dose given from day 14, and it was significantly higher (*p* < 0.001) than the response of mice immunized with the M recombinant protein alone (Figure 6B). The control group of mice remained seronegative with OD values less than 0.05 in both assays. These results indicate that both proteins produced by the recombinant strategy had the capacity to induce an immune response in animals despite the modification in the amino acidic sequence during the cloning process. This is strong evidence that the recombinant NP and M proteins had similar characteristics to the native viral proteins and, thus, they may be an advantageous alternative for the development of a new generation of diagnostic tools for BED.

### 3.6. Immunoreactivity Assays

**Figure 7:**
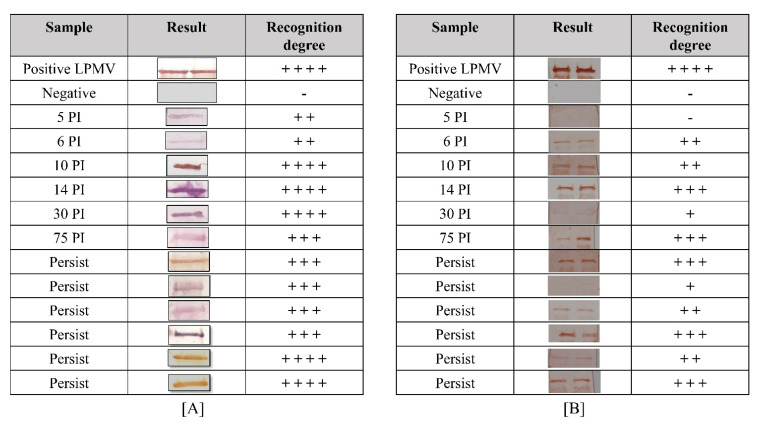
Immunoreactivity of recombinant proteins. (**A**) Immunoreactivity of the NP protein and (**B**) M protein with infected serum swine: (positive LPMV) hyperimmune serum (75 days post-infection) previously tested by indirect ELISA; (negative) Sweden serum; (5–75 PI) serum from experimentally infected swine after 5 days until 75 days post-infection; (persist) serum from naturally infected swine more than 90 days post-infection. The WB results were categorized by comparing the pixel intensity between positive control and serum samples with ImageJ program as follows: high grade had four marks (+ + + +) of recognition; +++ (moderate); ++ (slight); + (light); pig serum as a negative control with one negative mark (-).

For the determination of the immunodetection of the NP and M recombinant proteins, serum samples from experimentally and naturally PRV-infected pigs were tested by Western blot analysis (Figure 7). For the NP recombinant protein, the serum from the experimental infected pigs recognized the protein with a low degree from 5 until 6 days PI, with two positive marks (+ +). However, the recognition degree increased starting at 10 days PI with the maximum recognition (+ + + +) at 30 days PI. All serum samples from persistently infected pigs recognized the NP recombinant protein with three or more positive marks (representative results in Figure 7A).

For the M recombinant protein, the serum from experimentally infected pigs recognized the protein with high specificity after 6 days post-infection (PI). This recognition degree increased until 14 days PI, reaching three positive marks. An unexpected sample (30 days PI) slightly recognized the protein possibly due to the specific low response in this animal. Then, at 75 days PI, the recognition degree reached three positive marks again in all tested samples. For persistently infected animals (animals from farms and with more than 1 year PI), all samples recognized the protein with different degrees, demonstrating the presence of M antibodies in the animals for a long period of time (representative results in Figure 7B). This result indicates that the recombinant NP protein produced antibodies before the M protein and at high amounts in the infected animals. Finally, these recombinant proteins had necessary elements for the recognition of antibodies from infected pigs with different stages of PRV infection; therefore, they may have similar characteristics to those observed in the native protein, including conserved immunogenic epitopes.

## 4. Discussion

Since the blue eye disease (BED) appeared in Mexico, several studies focused on developing strategies for the diagnosis, prevention, and control of the disease. However, the viral disease remains endemic in the region denominated *Bajio Mexicano*, with sporadic outbreaks and new neurovirulent strains (2008–2015) with genetic differences being reported. These findings suggest that genetically and antigenically different PRV strains were circulating in the swine population, imposing challenges to diagnostic and vaccination efforts [20,26,38]. Thus, the virus is continuously adapting along the Mexican territory and prevails in the swine population. Moreover, classical techniques for BED diagnostics in laboratories, including hemagglutination inhibition tests, immunoperoxidase monolayer assays, and serum neutralizing tests that detect antibodies, are considered somehow obsolete, because they exhibit several disadvantages such as the use of chicken red blood cells, elution that occurs in a short incubation time, and a decrease in specificity [39]. In this context, significant efforts focused in producing new-generation strategies for the development of diagnostic methods or treatments have produced antigens in a yeast or bacterial heterologous system. Unfortunately, thus far, only the protein hemagglutinin-neuraminidase (HN) has been produced with favorable results [29,30]. Recently, the F protein of *Porcine orthorubulavirus* was studied, and several predictions regarding antigenicity were proposed including seven linear B-cell epitopes, six conformational B-cell epitopes, and twenty-nine T-cell MHC class I epitopes [40]. However, others structural proteins with interesting characteristics remain neglected, e.g., the nucleoprotein (NP) and the matrix (M) proteins. In the case of the NP protein, only nucleic acid-based identification methods (i.e., quantitative RT-PCR) were developed for the detection and quantification of the expression of the nucleoprotein gene for different strains [18]. In addition, Hernández et al., in 1998 [22], determined that the M and NP protein antibodies were produced by infected animals at 3 and 4 weeks post-infection during a 7-week study. As a consequence, the interest and relevance for producing new antigens from the PRV remain very attractive. Herein, the NP and M proteins were successfully produced using a bacterial heterologous expression system with the pETSUMO expression vector. Noticeably, others produced truncated versions of both the NP and the Matrix proteins from *Paramyxovirus* in *E. coli* by following a similar strategy with favorable results for the development of indirect ELISA and for protection against the disease following lethal viral challenges [27,41]. On the other hand, the production of recombinant proteins in pETSUMO generated large amounts of functional viral antigens with excellent immunogenic and antigenic properties, regardless of the 6-his and SUMO tag modifications [42,43]. In this study, the production yields for the NP and M recombinant proteins were 150 and 70 µg/mL, respectively, which were sufficient for mice immunization and WB analysis. Furthermore, the produced quantities of the recombinant proteins may still be adequate for pig immunization and vaccination studies based on previous studies in which authors used 400 µg per dose of the recombinant protein [44]. In antigenicity assays, we found that infected PRV animals produced anti-NP and anti-M protein antibodies starting from 5 days of PI and not after weeks of infection, as described in the only study that evaluated antibody production in infected swine [22]. The detection of recombinant proteins in the early stages of the PRV infection was possible because of the nanogram detection capabilities of the Western blot technique. Similarly, WB has allowed different authors to detect, with high specificity and from the first days of the infection, antibodies in serums of infected swine with viral recombinant proteins produced in *E. coli* [45]. Immunogenicity tests in mice described that both NP and M proteins had the capacity to induce an immune response in animals with detectable antibodies produced after 7 days post-immunization, with a sustained production that lasted during the 28 days of the experiment. Other investigations produced recombinant proteins in bacterial systems and used animal models (e.g., rabbits) to evaluate their antigenicity [37,38,39,40,41,42,43,44,45,46,47]. The results showed that the purified protein can react with positive serum (using the Western blot technique), displaying high antigenicity, which indicated that the protein could be used as an antigen for the detection of virus-specific antibodies in ELISA testing [46]. Finally, the PRV-NP protein had a similar domain organization as PIV5-N and Nipah virus-N proteins [37,47]. This fact allowed us to separate the structure into similar discrete subdomains, namely, the Narm, NTD, CTD, and Carm subdomains. The regions with the largest number of antigenic determinants were found in the NTD and CTD subdomains. On the other hand, the PRV-M protein had two similar domains that interact with other M protein domains to form dimers. These domains exhibit the principal antigenic determinants resembling the results found in the NDV-M protein [36]. These results substantiate our conclusions regarding the conservation of structural and antigenic determinants in the PRV based on similar findings with homologous proteins from other *paramyxoviruses* and between others PRV isolations reported in GenBank. In addition, amino-acid alignment comparisons between other PRV isolations reported in GenBank showed high amino acid identity, which is indicative that M and NP protein have been conserved for long time. Varsanyi et al., 1985, mentioned that the particular case of the conserved regions of the *Paramyxovirus* family that exhibit conserved sequences is highly likely to preserve their tertiary structure so that they can maintain their biological function. [48]. Therefore, the probability of finding failures in the development of diagnostic test with these antigens is low.

## 5. Conclusions

Finally, in this study and for the first time, we successfully produced nucleoprotein (NP) and matrix (M) proteins from PRV by using a recombinant strategy in the *E. coli* heterologous expression system. These proteins were fused to 6-his and SUMO tags at the N-terminus to facilitate the purification process and to increase their solubility, respectively. Despite these modifications, both proteins conserved antigenic characteristics and were recognized by infected swine serum since the early stages of the infection until persistence (more than 90 days PI). The present study showed that recombinant NP and M proteins induced immune responses in an animal model (mice) with detectable antibodies starting from the 7th day until 28 days post-immunization. Additionally, the predicted tertiary structure showed that both proteins had antigenic determinants that were conserved with members of the *Paramyxoviridae* family. Our results pave the way for developing biotechnological tools based on these proteins for the control and prevention not only of the blue eye disease but also other infections caused by paramyxoviruses.

## Figures and Tables

**Table 1:** Primers used to amplify the open reading frames of the M and NP gene of PRV.

Gene	Forward Primer	Reverse Primer	Amplicon
M	ACTGCACAACTTCAACCATTTCC	GCTACTACTTACGGAAAGGATTCCAG	1066
NP	CTCCAGGATAGAGGAGCCGA	CTACTAAAGCTGCTGAAACATGGC	1563

## Data Availability

Not applicable.
